# Surface Loading Dictates Triplet Production via Singlet Fission in Anthradithiophene Sensitized TiO_2_ Films

**DOI:** 10.1021/acs.jpcc.4c04284

**Published:** 2024-08-12

**Authors:** Melissa K. Gish, Katherine Snell, Karl J. Thorley, John E. Anthony, Justin C. Johnson

**Affiliations:** † Materials, Chemistry and Computational Sciences Directorate, National Renewable Energy Laboratory, Golden, Colorado 80401, United States; ‡ Department of Chemistry, University of Kentucky, Lexington, Kentucky 40506, United States

## Abstract

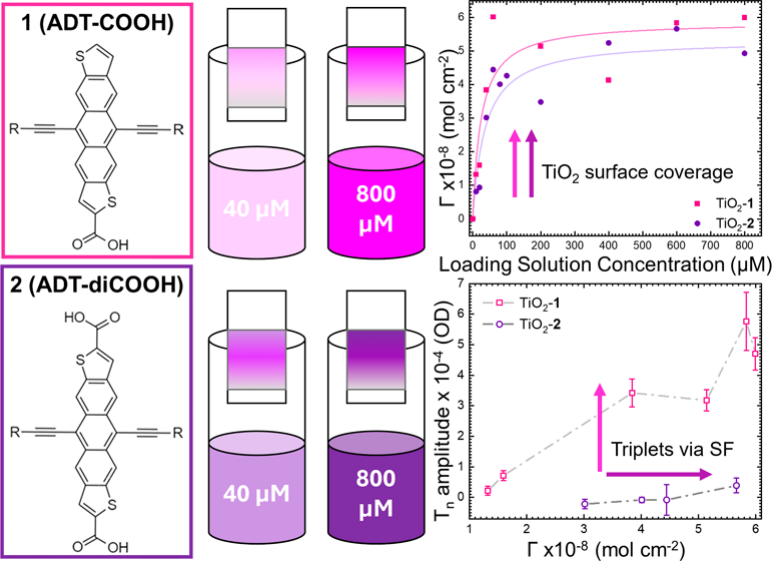

Singlet fission, the process of transforming a singlet excited state into two lower energy triplet excited states, is a promising strategy for improving the efficiency of dye-sensitized solar cells. The difficulty in utilizing singlet fission molecules in this architecture is understanding and controlling the orientation of dyes on mesoporous metal oxide surfaces to maximize triplet production and minimize detrimental deactivation pathways, such as electron injection from the singlet or excimer formation. Here, we varied the concentration of loading solutions of two anthradithiophene dyes derivatized with either one or two carboxylic acid groups for binding to a metal oxide surface and studied their photophysics using ultrafast transient absorption spectroscopy. For the single carboxylic acid case, an increase in dye surface coverage led to an increase in apparent triplet excited-state growth via singlet fission, while the same increase in coverage with two carboxylic acids did not. This study represents a step toward controlling the interactions between molecules at mesoporous interfaces.

## Introduction

Molecular-semiconductor systems combine effective light absorption within the solar spectrum with the spatial separation of electrons and holes at an interface. Primarily used in photovoltaic and photo(electro)catalytic systems, efficiencies of these devices are thermodynamically constrained to the detailed-balance limit.^1,2^ One of the assumptions of this limit is the generation of a single electron–hole pair with each incoming photon. Singlet fission (SF) molecules can generate two electron–hole pairs with a single photon with high efficiencies and remain a strategy to circumvent these constraints.^3,4^ SF is governed by a set of well-defined molecular design principles^5^ that are comprehensively -studied in solution,^6,7^ in crystals,^8−11^ and in neat films.^12−16^

Adhering to these design criteria is not so simple in realistic architectures that are also engineered to harvest charges, i.e., dye-sensitized solar cells (DSSCs).^3,17^ Typical DSSCs employ chromophores adsorbed to a mesoporous metal oxide surface (e.g., TiO_2_), where photoexcitation leads to rapid excited-state electron injection from singlet and triplet states with appropriate driving force.^16,18−27^ DSSCs rely on binding of carboxylic acid- or phosphonic acid-functionalized chromophores at the oxide surface via dip coating, where surface coverage depends on the concentration of loading solution.^21,26,28^ This makes fabrication of DSSCs facile; however, it is difficult to control intermolecular interactions to promote processes such as SF and outcompete singlet excited-state electron injection.

We and others have performed fundamental studies that revealed problems associated with competing processes with SF, including charge transfer from the photoexcited singlet and excimer formation.^18,19,21,25,29^ These are inherent to molecules at charge-accepting interfaces and suggest additional design criteria are necessary, mostly surrounding intermolecular and molecule-semiconductor distances and geometries. Applying these principles requires additional fundamental studies.

**Figure 1:**
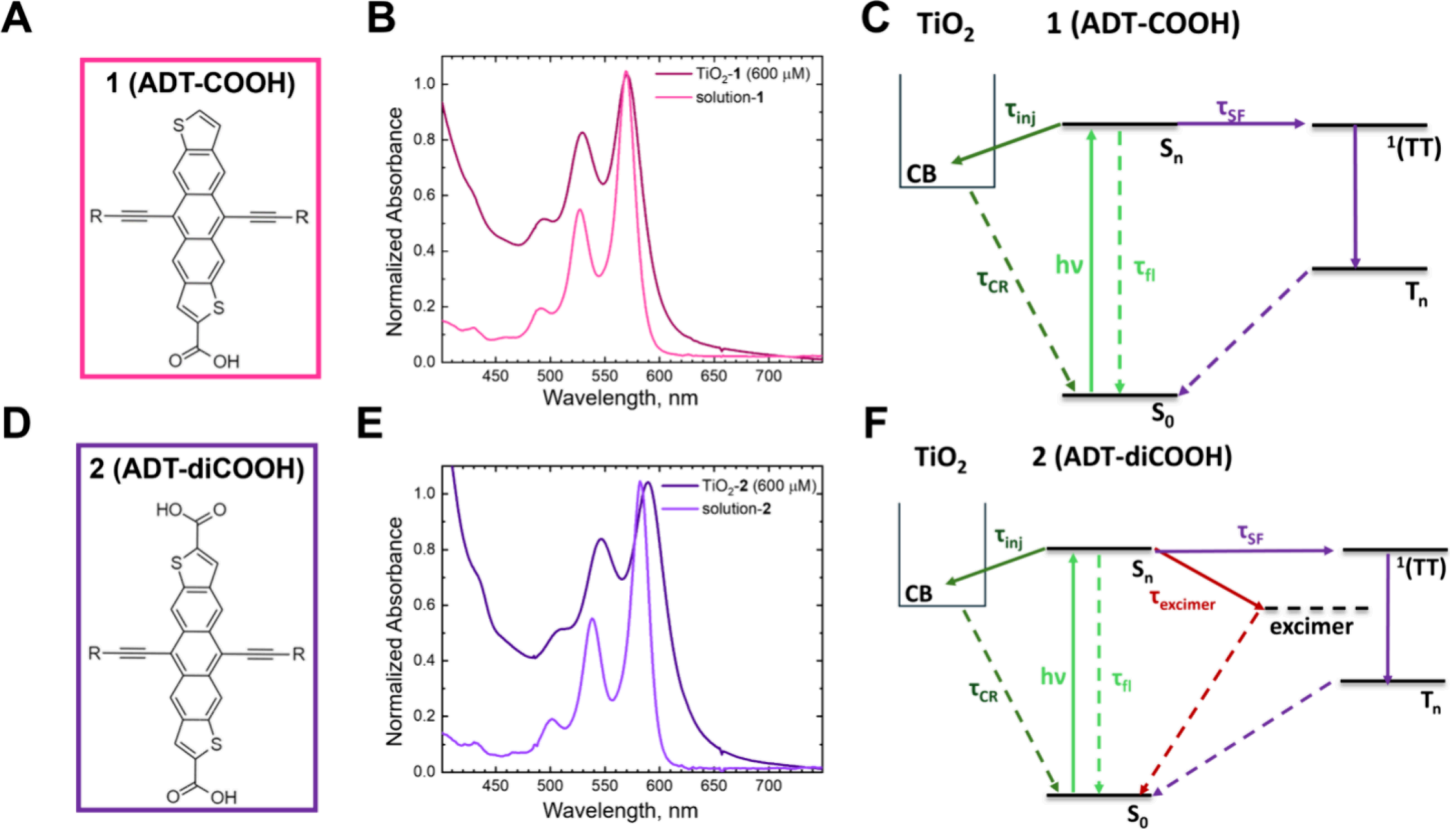
Anthradithiophene functionalized with one ((A) ADT-COOH and **1**) or two ((D) ADT-diCOOH and **2**) carboxylic acid groups. Normalized steady-state absorption spectrum of **1** ((B) light pink) or **2** ((E) dark purple) in THF solution (light pink) and adsorbed to mesoporous TiO_2_ ((B) TiO_2_-**1**, dark pink and (E) TiO_2_-**2**, dark purple). Kinetic scheme of possible pathways following photoexcitation of **1** (C) or **2** (F) adsorbed to TiO_2_. In panels A and D, R = triisobutylsilyl.

Here, we vary the concentration of loading solutions of an SF molecule, anthradithiophene (ADT), functionalized with one or two carboxylic acid groups, **1** and **2**, respectively, for binding to a mesoporous TiO_2_ surface (Figure 1). The results build upon prior work showing that the diacid in polycrystalline thin films undergoes hydrogen bonding that may encourage strong and direct π–π stacking even at the lowest loading concentrations.^13^ In contrast, the interactions between monoacids in aggregated forms may be more balanced between π–π stacking and hydrogen bonding, which would be eliminated after the covalent binding of the monoacid to the TiO_2_ surface. Despite similar surface coverages, we find that an increase in loading solution concentration for the monoacid (**1**) leads to an increase in apparent triplet excited-state growth, while the same procedure for the diacid (**2**) does not and instead leads to increased formation of excimers.

Figure 1C,F shows the possible pathways after photoexcitation for TiO_2_-**1** and TiO_2_-**2**. Excitation of the chromophore creates a singlet excited state (S_*n*_), which has a sufficient driving force to undergo electron injection into the conduction band (CB) of the TiO_2_. In competition with charge transfer and dependent on intermolecular interactions, SF occurs through the creation of a correlated triplet pair ^1^(TT) to form triplet excited states (T_*n*_). A third pathway is introduced in TiO_2_-**2** with the addition of a second unbound carboxylic acid toward excimer formation, preventing efficient SF. We provide a full photophysical characterization of these systems and hypothesize about the competition between singlet fission and excimer formation, which are in principle separately controllable pathways.

## Experimental Methods

### Steady-State Absorption

Steady-state absorption spectra were collected by using a UV–visible–NIR absorption spectrophotometer (Agilent Technologies, model 8453 A) with an air blank to correct the baseline.

### Sample Preparation

Synthesis of the compounds used in this study is described in ref (13). Mesoporous TiO_2_ films were prepared on glass substrates that had been ozone cleaned. The TiO_2_ paste consisted of 20 nm anatase TiO_2_ nanoparticles in a suspension with terpineol and ethylcellulose purchased from Sigma-Aldrich and used without further purification. The paste was doctor bladed onto the glass substrates marked with one layer of scotch tape to control the placement and thickness as described in ref (20), resulting in ∼1 cm^2^ active area. The films were sintered at 500 °C and slowly cooled back to room temperature. Solutions of known concentrations of **1** and **2** in THF solution were prepared, and 1.3 cm × 2.5 cm TiO_2_ slides were completely submerged in the solutions of known concentrations overnight. After being soaked, the films were soaked in neat THF to remove unbound molecules. All transient experiments were conducted in an air-free cell assembled in a N_2_ glovebox to prevent effects from the presence of oxygen.

### Time-Resolved Photoluminescence

Time-resolved photoluminescence data were collected using a Hamamatsu Streak Camera (300–900 nm, C10910-04) with an NKT supercontinuum fiber laser (SuperK EXU-6-PP) routed to an acousto-optic tunable filter (SuperK Select). The samples were photoexcited at 515 nm with a repetition rate of approximately 3 MHz.

### Transient Absorption Spectroscopy

Transient absorption data were collected in a pump–probe configuration using a Coherent Libra Ti:sapphire laser with an 800 nm fundamental (150 fs pulse width, 1 kHz rep rate). The pump pulse was generated in an optical parametric amplifier (TOPAS-C, Light Conversion). Pulse energies were 30 nJ/pulse, unless otherwise noted. The probe pulse was generated via supercontinuum generation in a thin sapphire (λ_probe_ = 440–850 nm) or a thick sapphire (λ_probe_ = 850–1500 nm) window. A mechanical delay stage was used to delay the probe relative to the pump, and the probe was focused at the sample. The pump was spatially overlapped with the probe at the sample to maximize the signal. A small amount of probe was picked off before the sample and directed to a reference detector to maximize signal-to-noise ratio. A fiber-coupled multichannel spectrometer with a CMOS sensor (Ultrafast Systems) was used to monitor changes in the probe spectrum. Collection software (Helios) was provided by Ultrafast Systems. Data were chirp corrected using Surface Xplorer (Ultrafast Systems) and analyzed in Origin (OriginLabs).

### Data Analysis

Global fitting was performed in MATLAB with an in-house written script to fit the entire spectrum to a three-component parallel decay model where S_1_ could go to T_1_ or to the cation. The peak amplitudes for S_1_ and T_1_ were extracted from the fits, and the ratio was taken to confirm our analysis of the raw data as a function of surface loading. The Density-Based Spatial Clustering of Applications with Noise (DBSCAN) analysis was performed in MATLAB along with the grid analysis.^45^

## Results and Discussion

The behavior in solution of the compounds used in this study has been reported previously.^13^ Briefly, the UV–visible absorption spectra show a vibronic progression typical of these molecules with a 15 nm (∼45 meV) red shift between the **1** and **2** derivatives, as shown in Figure 1B,E. The singlet energy shifts slightly from 2.14 to 2.11 eV with the addition of the second carboxylic acid with typical fluorescence lifetimes of approximately 20–25 ns. For **1**, adsorption to TiO_2_ results in slight broadening of the peaks, but no significant change in the 0–0 peak position (Figure 1B). In **2**, however, adsorption to TiO_2_ causes an approximately 10 nm (∼35 meV) shift in the 0–0 position (Figure 1E), suggesting that some intermolecular electronic interaction between molecules is occurring when immobilized at the metal oxide interface.

**Figure 2:**
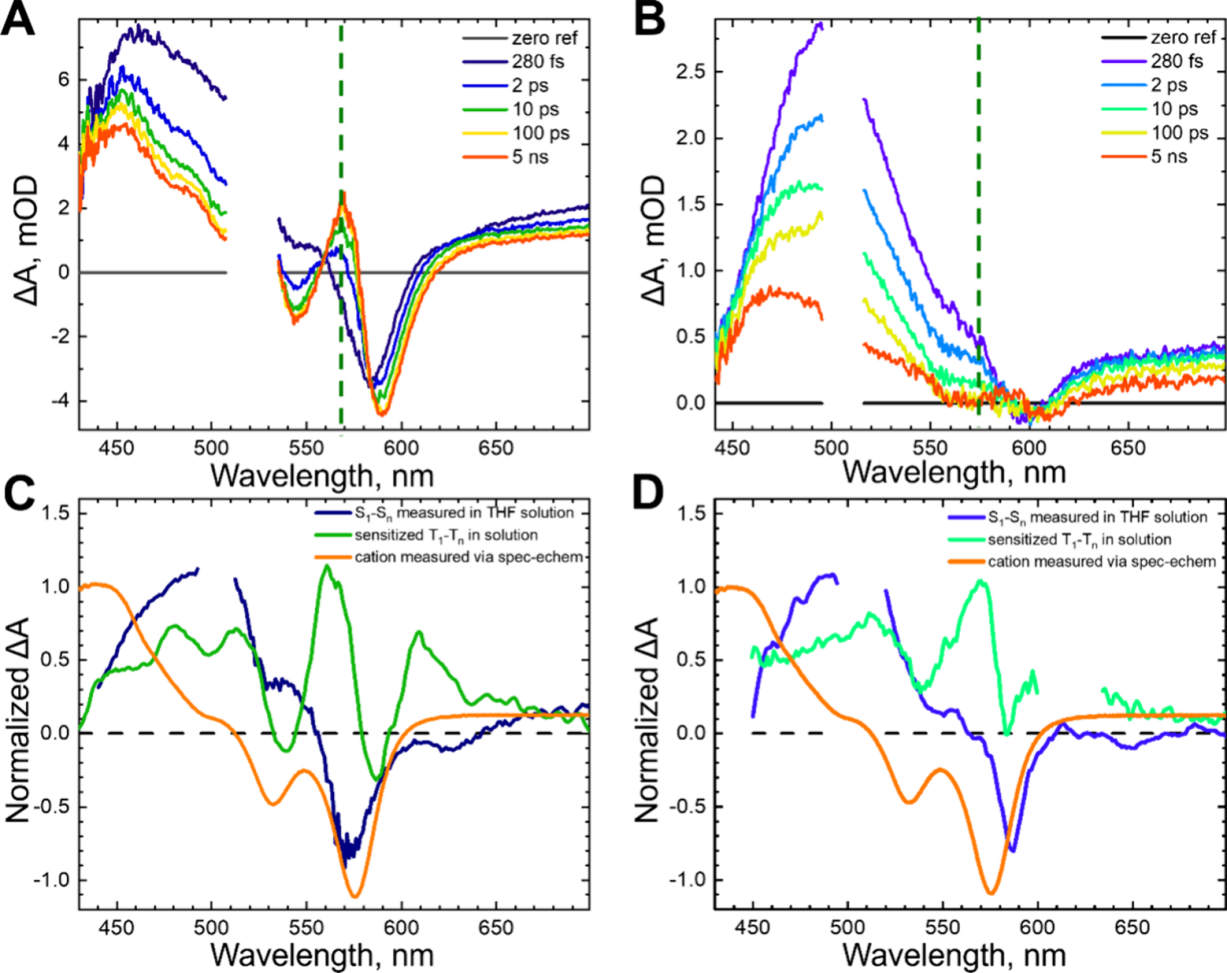
Transient absorption data of (A) TiO_2_-**1** and (B) TiO_2_-**2** at high surface coverages ([dye] = 600 μM) photoexcited at 515 or 505 nm, respectively (30 nJ/pulse). Pump–probe delays are shown in the legend. The green dotted line indicates the peak wavelength of the relevant triplet excited-state spectrum. Panels C and D show representative component spectra for the singlet excited state (S_1_–S_*n*_) (**1**: dark blue, **2**: blue), triplet excited state (T_1_–T_*n*_) (**1**: green, **2**: light green), and the radical cation (**1**,**2**: orange). They are normalized for clarity. The data in panels C and D are reproduced from ref (29). Available under a CC BY-NC 3.0 license. Copyright 2021 Gish et al.

Representative transient absorption spectra of the sensitized films with high surface coverages ([dye] = 600 μM) after 505 nm photoexcitation are shown in Figure 2A,B for TiO_2_-**1** and TiO_2_-**2**, respectively. Additional transient absorption data for low and high surface coverages are available in the Supporting Information. At 280 fs after photoexcitation, TiO_2_-**1**(600 μM) exhibits photoinduced absorptions (PIAs) at <500 and >620 nm with a ground-state bleach (GSB) at 580 nm. This initial spectrum in Figure 2A resembles the isolated molecule in THF solution as seen in the dark blue trace in Figure 2C, which we assign to the singlet excited state (S_1_–S_*n*_) as charge transfer and SF does not occur in dilute solution.^13,29^ Over the course of 5 ns, the spectra evolve to contain a 450 nm PIA with a shoulder at 490 nm, a PIA centered at 570 nm, and enhanced ground-state bleaching at 540 and 590 nm. Based on our control experiments (Figure 2C), the 5 ns spectrum appears to be a superposition of the cation measured via spectroelectrochemistry (orange) and the triplet excited state measured via anthracene sensitization (green). Low surface coverage experiments for TiO_2_-**1**(40 μM) (Figure S2A) exhibit similar dynamics; however, at 5 ns, the dominant species in the visible is the cation with a smaller contribution from the feature associated with the triplet excited state. Fitting the kinetics at the peak of the triplet feature (565–575 nm, Figure S2D), for TiO_2_-**1** surface loadings >1.5 × 10^–8^ mol·cm^–2^ (≥40 μM loading solution) using shared time constants, reveals a biexponential rise of approximately 1.5 and 20 ps.

TiO_2_-**2**(600 μM) reveals markedly different behavior in the transient absorption data (Figure 2B). The initial spectrum at 280 fs after photoexcitation also resembles that of the isolated molecule in a THF solution (Figure 2D, blue), although the GSB is not as prominent. The spectra through the 5 ns window evolve as a blue shift in the 500 nm PIA to one centered around 460 nm, and there is a small PIA centered at 590 nm that appears at later pump–probe delays. The GSB features become slightly more prominent, though not to the same extent as TiO_2_-**1**(600 μM). While the TiO_2_-**2** TA data appear to have contributions from both the cation and triplet (Figure 2D, orange and light green, respectively), there is an additional component at 475 nm that arises due to the presence of another species that we tentatively assign as an excimer (Figure S4D). Transient absorption data for varying surface coverages of TiO_2_-**2** are shown in Figure S4 and demonstrate a shift in behavior from mostly singlet excited-state electron injection to intermolecular excimer formation.

**Figure 3:**
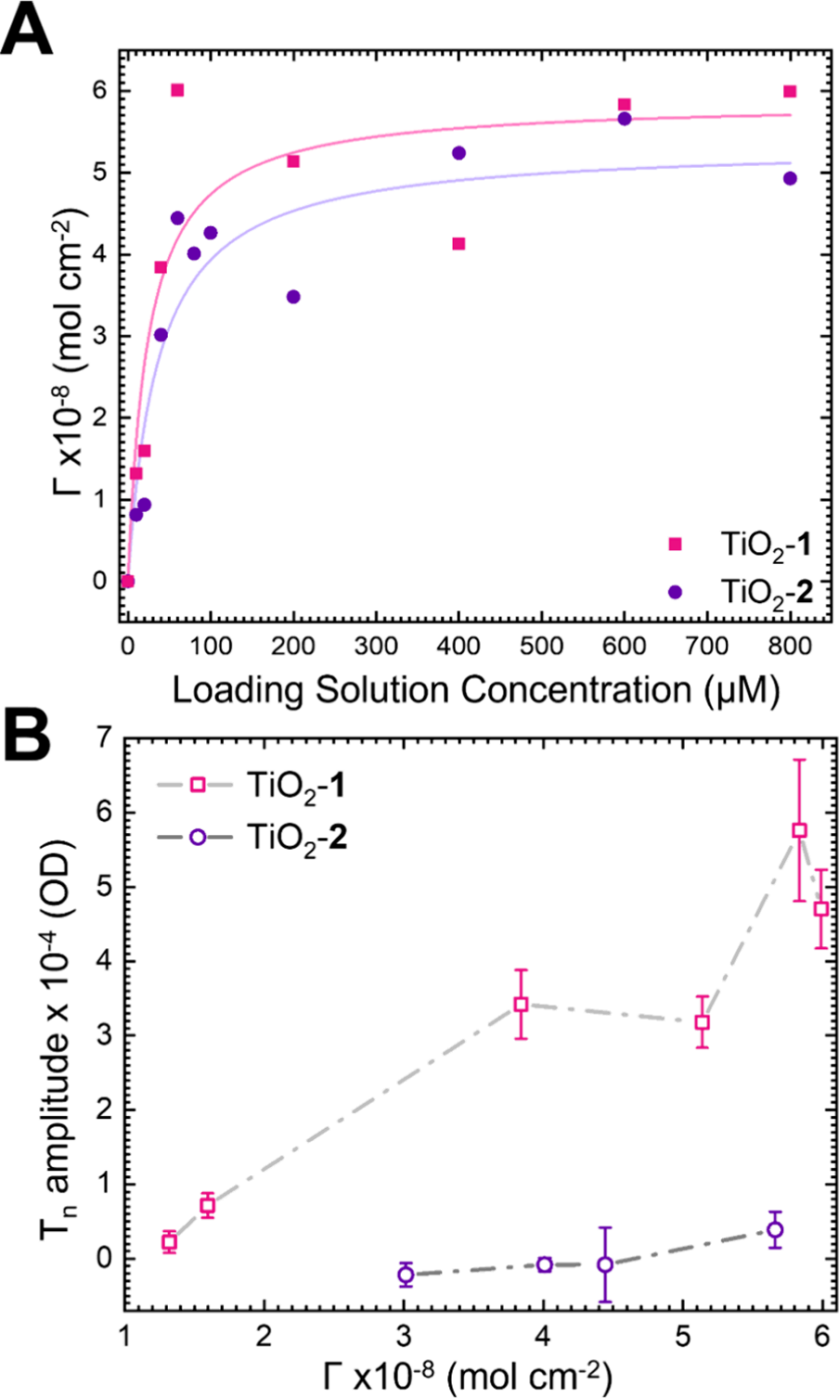
(A) Surface coverage of **1** (pink squares) and **2** (purple circles) on TiO_2_ as a function of the concentration of the loading solution. (B) T_*n*_ amplitudes of **1** (pink hollow squares) and **2** (purple hollow circles) adsorbed to TiO_2_ as a function of surface coverage.

The surface coverages for TiO_2_-**1** (pink squares) and TiO_2_-**2** (purple circles) as a function of loading solution are shown in Figure 3A. These adsorption isotherms were calculated by varying the concentration of the loading solution from 10 μM through 800 μM, measuring the absorption spectra, and estimated with the equation Γ = A(λ)/ε(λ)/1000.^21,26,30^ The molar absorptivities (ε(λ)) used in this equation were measured to be 21,412 and 20,569 mol^–1^·cm^–1^ at the 0–0 peak for **1** and **2**, respectively. The steady-state absorption spectra as a function of loading solution concentration are shown in Figure S1. Both **1** and **2** follow similar trends where the surface coverage increases significantly up to 200 μM and begins to level out between 400 and 800 μM. The maximum surface coverages are approximately 5 × 10^–8^ mol·cm^–2^ for both derivatives.

Figure 3B shows the maximum triplet excited-state (T_*n*_) amplitude as a function of the surface coverage. Because of the complex kinetic scheme and overlapping spectral features in this system, as demonstrated in Figure 2C,D, it is difficult to quantify a triplet yield. Instead, we measured varying surface coverages at the same excitation power (Figure S2D) and integrated over peak T_*n*_ signal wavelengths from 565 to 575 nm, taking the average signal where the amplitude is no longer changing (τ_probe_ > 50 ps). The triplet excited-state rise kinetics do not change as a function of excitation power (Figure S3), but we used 30 nJ for this analysis to avoid the possibility of accelerated cation recombination (Figure S10) that may artificially increase the triplet excited states signal.^31,32^ A clear increase in T_*n*_ amplitude is observed for TiO_2_-**1**, while this amplitude remains at or near zero for TiO_2_-**2**, despite showing similar trends in surface coverage as TiO_2_-**1**. Global analysis was performed to confirm the relative increase in triplet excited-state contribution vs dye coverage (Figures S5 and S6). The fit employed a simplified kinetic scheme that includes parallel S_1_ → TT and S_1_ →cation decay pathways and resulted in decay associated spectra (DAS) that we use to determine the relative yield of triplets (DAS are shown in Figure S5). The peak of the secondary amplitude (A_T_) was divided by the peak of the initial amplitude (A_S1_) for each concentration. The result is shown in Figure S6, which closely follows the analysis of T_*n*_ amplitude derived from considering the raw signals (Figure 3B).

Time-resolved photoluminescence (TRPL) spectra at the maximum surface coverages show that TiO_2_-**1** maintains an emission profile similar to that of the fluorescence measured in solution that we have reported previously (Figure S7A).^13^ It contains a vibronic progression mirroring steady-state absorption and undergoes minimal change throughout the observation window. The TiO_2_-**2** emission profile, however, is significantly red-shifted and featureless compared to that measured in solution (Figure S7B). This change in the emission spectrum has been widely assigned to excimer formation in many organic systems.^21,23,33−37^ Based on the immediate appearance of the excimer feature in the TRPL data, we are confident in assigning an excimer contribution to the transient absorption data of TiO_2_-**2**. This assignment was confirmed based on a persistent PIA feature (Figure S4D) present in a saturated solution of **2** in THF that resembles neither the singlet excited-state spectrum nor the measured triplet or cation spectra. For TiO_2_-**2**(40 μM) (Figure S4A), the data look similar to the high surface coverage case in Figure 2D with a lower signal intensity due to fewer molecules on the surface.

**Figure 4:**
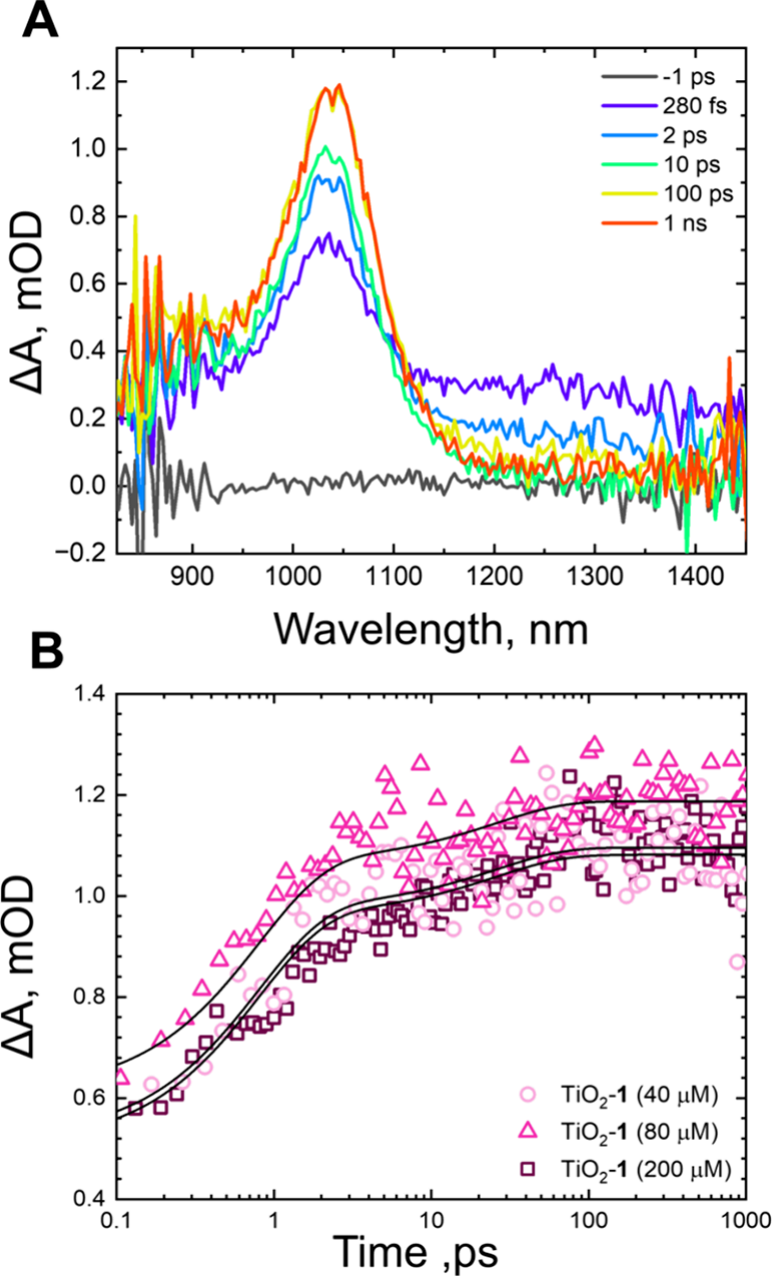
(A) NIR transient absorption spectra of TiO_2_-**1**(200 μM) after photoexcitation at 505 nm (30 nJ/pulse). Pump–probe delays are shown in the legend. (B) Transient absorption kinetics at a probe wavelength of 1030 nm of TiO_2_-**1**(40 μM) (light pink circles), TiO_2_-**1**(80 μM) (pink triangles), and TiO_2_-**1**(200 μM) (dark pink squares). Fits are shown as black lines.

Transient absorption spectra were collected in the NIR spectral region for the low and high surface coverages for TiO_2_-**1** (Figure 4, Figure S8) and TiO_2_-**2** (Figure S9). The NIR region of the spectrum is remarkably less congested than the visible with clear and separated contributions from the singlet excited state (λ_max_ = 1300 nm) and the cation (λ_max_ = 1030 nm).^29^ In all cases, the cation is present at the earliest pump–probe delay, suggesting that there is an ultrafast component to excited-state electron injection within our instrument response. The cation signal continues to rise and behaves similarly for TiO_2_-**1** and TiO_2_-**2** (Table S1). This is not surprising as the driving forces for excited-state electron injection from the singlet state in both dyes are sufficiently close [∼−0.8 eV] that the electron transfer rates into the conduction band of TiO_2_ should be comparable. Multiexponential electron injection kinetics is a characteristic of DSSCs. This behavior arises due to excitation into an unequilibrated, or “hot” singlet excited state overlapped with a density of states in the TiO_2_ conduction band leading to charge injection within our instrument response.^38^ Localized trap states at the TiO_2_ surface also leads to a distribution of electron injection time constants.^39^

The kinetics associated with the singlet excited state (λ_probe_ = 1300 nm) also display fast decay lifetimes; however, there are significant differences between TiO_2_-**1** (Figure S8) and TiO_2_-**2** (Figure S9, Table S2). TiO_2_-**1** at all surface coverages exhibits time constants of 0.8 and 6.1 ps, which is the approximate average of the cation growth and triplet excited-state growth kinetics. TiO_2_-**2** with high and low surface coverages has time constants of 2.0 and 163 ps. This long lifetime is substantially slower, and with the visible spectral range data, we assign this 163 ps time constant in TiO_2_-**2** to the formation of excimers.

The stark differences in behavior between TiO_2_-**1** and TiO_2_-**2**, despite their highly similar molecular structures and properties, must be due to the way they are packing on the surface. Previous work from our group has shown that small differences in intermolecular interactions can lead to significant impacts on singlet fission dynamics.^12,13,15,27^ In fact, neat thin-film configurations of **1** and **2** demonstrate the opposite behavior from the current work where triplets form readily via singlet fission in thin films of **2** due to extended hydrogen bonding networks between molecules. This is not possible in neat thin films of **1** because the single carboxylic acid prevents anything beyond dimer-like interactions. On a mesoporous metal oxide film, however, the molecules bind to the surface through the carboxylic acid group. This eliminates the possibility that **1** can participate in hydrogen bonding and their self-assembly leads to favorable orientations for SF. Meanwhile, the presence of a secondary carboxylic acid in TiO_2_-**2** likely predisposes molecules to configurations less favorable for SF. We postulate that the unbound carboxylic acid in **2** engages in lateral hydrogen bonding,^40^ tilting the molecules toward each other creating a favorable interaction for excimer formation. Several binding modes for carboxylic acid groups at the TiO_2_ surface are possible, including an ester-like linkage where one oxygen within the carboxylate remains unbound.^41^ Additionally, lateral hydrogen bonding between bound dicarboxylic functionalized acenes has been observed on quantum dot surfaces.^42^ Together, these studies suggest that it is feasible that molecules of **2** may tilt toward each other on TiO_2_. The competition between excimer formation and triplet formation from SF is well-known to hinge on somewhat subtle changes in intermolecular geometries.^12,15,35,43^ Due to the rationalized differences in triplet forming behavior between solution, thin films, and at a metal oxide interface for both molecules, we are confident in our assignment of triplet generation via SF and have ruled out intersystem crossing,^44^ which would be expected to depend only minimally on intermolecular interactions.

The data suggest several regimes of molecular interactions with either each other or the TiO_2_ surface. At low surface coverages, the molecules are more likely to be isolated from one another, making the dominant pathway singlet electron injection (Figure 1C,F). As surface coverage increases, the density of molecules on the surface increases, making intermolecular interactions more likely.^27^ Since the cation signal remains consistent with an unchanged rate as surface coverage increases (Figure 4), it is likely that the binding configuration for each molecule is roughly constant throughout the concentration series, and it is the intermolecular interactions that change the other photophysical behavior. The monoacid in TiO_2_-**1** self-assembles in a stochastic fashion, following the Langmuir model, which must lead to configurations amenable to singlet fission that become more likely as coverage increases. To verify this hypothesis, we performed a cluster analysis of the results of a stochastic dye binding simulation vs loading of a surface (Figure S11). The results reveal that dimers (i.e., a bound dye with a nearest neighbor) become probable with <20% surface loading (Figure S12), in contrast to the shallower rise in triplet yields observed experimentally shown in Figure 3B. The probability of clusters of 3, 4, and 5 dyes rises with increased surface coverage more similarly to the experimental triplet yield trend, leading us to conclude that clusters of more than two molecules facilitate the formation of long-lived triplets. This aligns with our prior results on neat ADT films, where chains of just two molecules did not sustain long-lived triplets while longer chains did.

Several questions remain about how the photophysical behavior connects to local geometries. One is how is the excimer competitive with either charge injection or singlet fission, if its formation time is roughly 200 ps compared with <10 ps for the other processes? One possibility is that, as the aggregated islands of **2** grow with concentration, energy transfer within the ensemble causes a downhill cascade toward the pair of molecules most likely to undergo excimer formation. As energy from the initially excited localized singlet is lost through the cascade, both charge-transfer to TiO_2_ and singlet fission become slower (i.e., smaller driving force) and excimer formation becomes the dominant pathway. The initiation of this process may compete with singlet fission or charge separation on the picosecond time scale, but its completion may not be spectroscopically distinguished until later. Additionally, **1** has an excited-state dipole oriented toward the TiO_2_ surface, while the addition of the second carboxylic acid in **2** removes this directionality. While the driving forces are similar, the changes in the excited-state dipole may increase competition between excimer formation and electron injection.

Another question is whether triplet lifetime elongates as aggregates of **1** get larger, and if so, does that lead to better opportunities for charge transfer from the triplet state? Questions regarding this second step in the process of a singlet fission DSSC were addressed in our prior work. For unbiased TiO_2_ and ADT, it is highly unlikely that triplets will dissociate due to an energy barrier for electron transfer, and answering these questions will require further work on other charge-accepting electrodes.

In conclusion, we have established that small changes in molecular structure lead to significant differences in the photophysics at a mesoporous TiO_2_ interface, where a single carboxylic acid on an anthradithiophene promotes triplet formation via SF and a second carboxylic acid group orients in a way favorable to excimer formation. We also observed a surface coverage dependence for TiO_2_-**1** on the relative yield of the SF-generated triplet excited states. We postulate that this is due to the self-assembly of the molecules at the TiO_2_ surface. Experiments to quantitatively characterize interactions of these molecules on surfaces are ongoing. This study demonstrates the importance of careful molecular design when self-assembly is reliant on an interface.

## Supporting Information Available

Supporting Information includes additional transient absorption spectra and kinetics for different loading concentrations (PDF)
