# Supplementary material for: Surface Loading Dictates Triplet Production via Singlet Fission in Anthradithiophene Sensitized TiO2 Films
Source: J Phys Chem C Nanomater Interfaces. 2024 Aug 12;128(33):13944–51. doi: 10.1021/acs.jpcc.4c04284 (PMC11345824; doi:10.1021/acs.jpcc.4c04284)
Supplement: Supplementary file 1 — jp4c04284_si_001.pdf [file jp4c04284_si_001.pdf]

Supporting Information for:

Surface Loading Dictates Triplet Production via Singlet Fission in Anthradithiophene Sensitized TiO_2 Films

Melissa K. Gish,[1] Katherine Snell[1,‡] Karl J. Thorley,[2] John E. Anthony,[2] Justin C. Johnson[1]**

1. Materials, Chemistry and Computational Sciences Directorate, National Renewable Energy Laboratory, Golden, Colorado 80401, United States.
2. Department of Chemistry, University of Kentucky, Lexington, Kentucky, 40506, United States.

AUTHOR INFORMATION

Corresponding Author

*E-mail: melissa.gish@nrel.gov, justin.johnson@nrel.gov

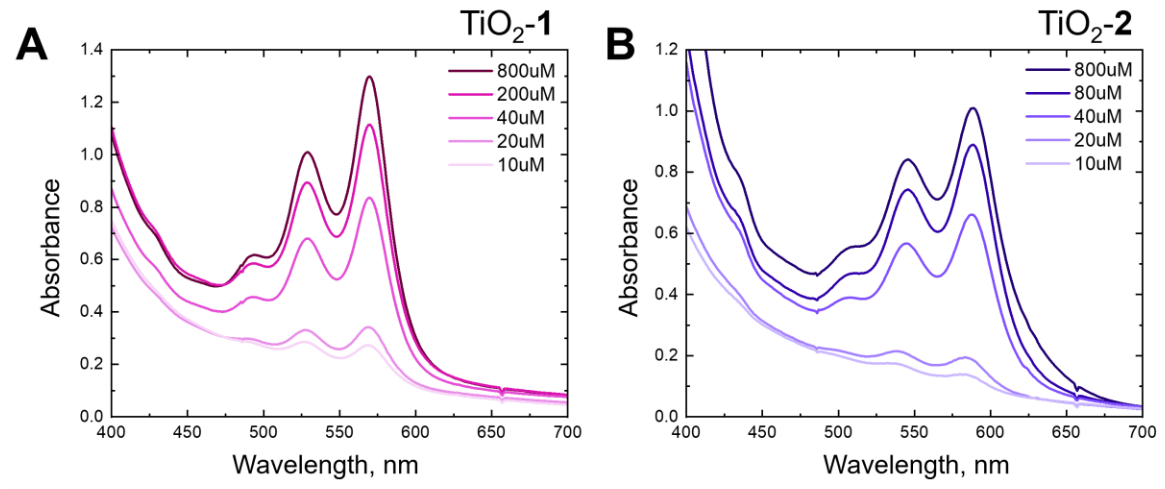

Figure S1. Steady state absorbance spectra of A. TiO_2-**1** and TiO_2-**2** at varying loading solution concentrations.

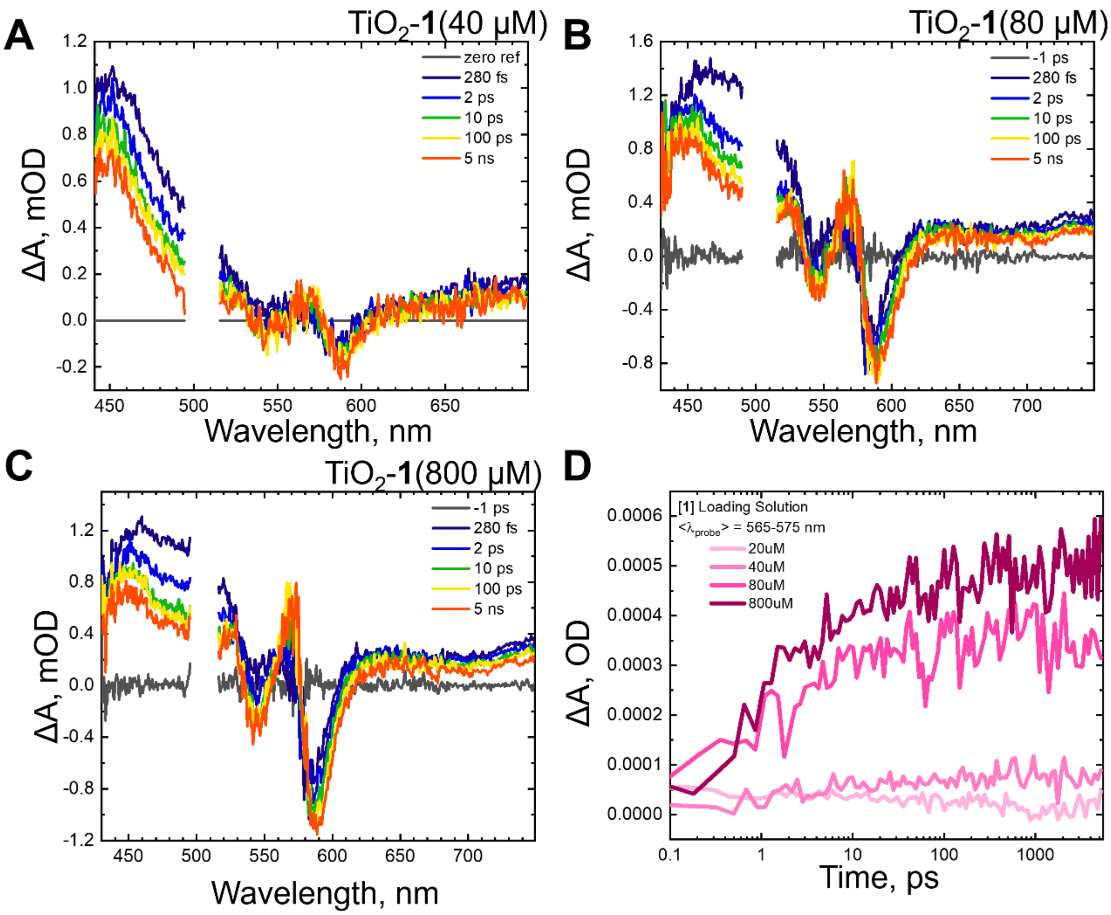

Figure S2. Transient absorption spectra of **A.** TiO_2-**1**(40 µM), B. TiO_2-**1**(80 µM), **C.** TiO_2-**1**(800 µM) after 515 nm (30 nJ/pulse) excitation. Pump-probe delays are shown in the legend. **D.** Transient absorption kinetics of TiO_2-**1** at varying surface coverages averaged at the peak wavelengths of the triplet excited state feature (565-575 nm).

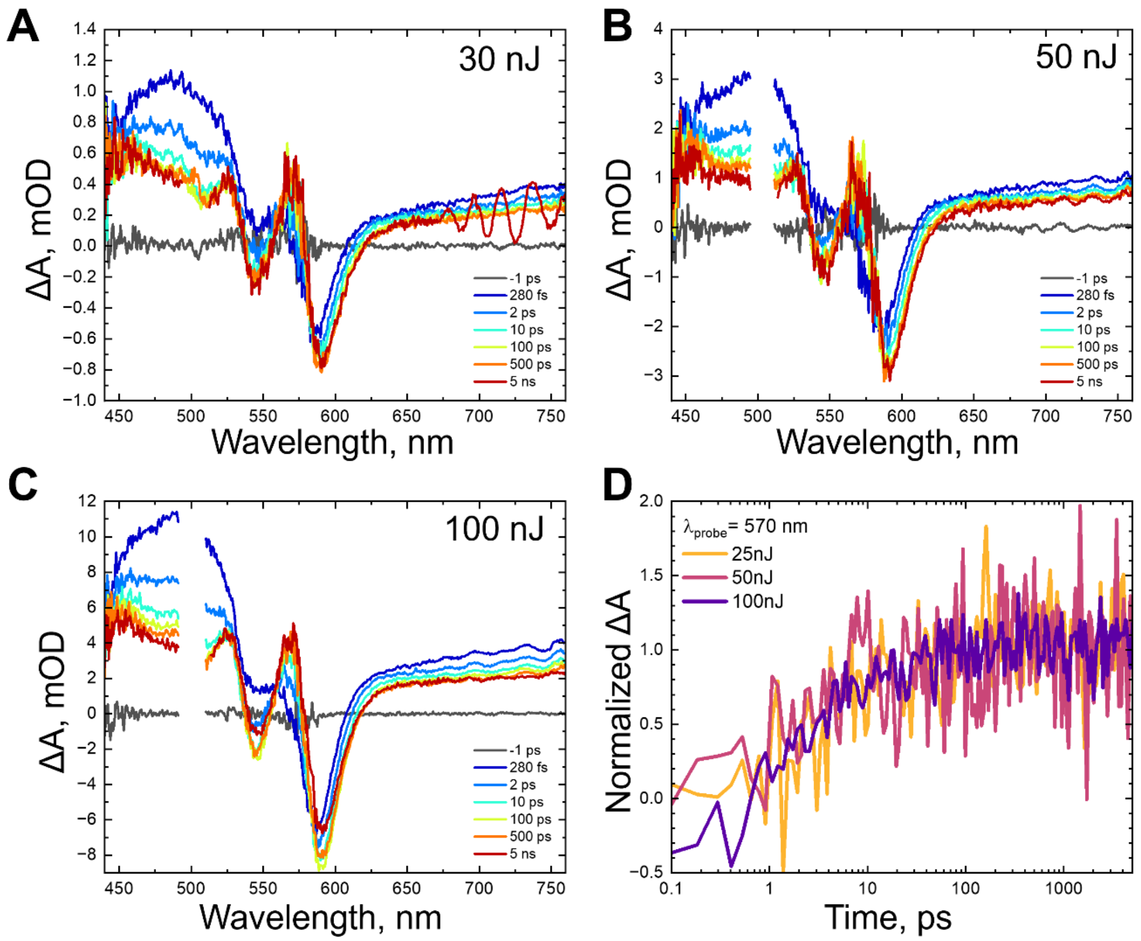

Figure S3. Transient absorption spectra of TiO_2-**1**(200 μM) after 505 nm excitation at varying powers of **A.** 30 nJ/pulse B. 50 nJ/pulse and **C.** 100 nJ/pulse. Pump-probe delays are shown in the legend. **D.** Normalized transient absorption kinetics of TiO_2-**1** at varying powers probed at 570 nm, the triplet excited state feature. The behavior of this feature does not change with increasing power.

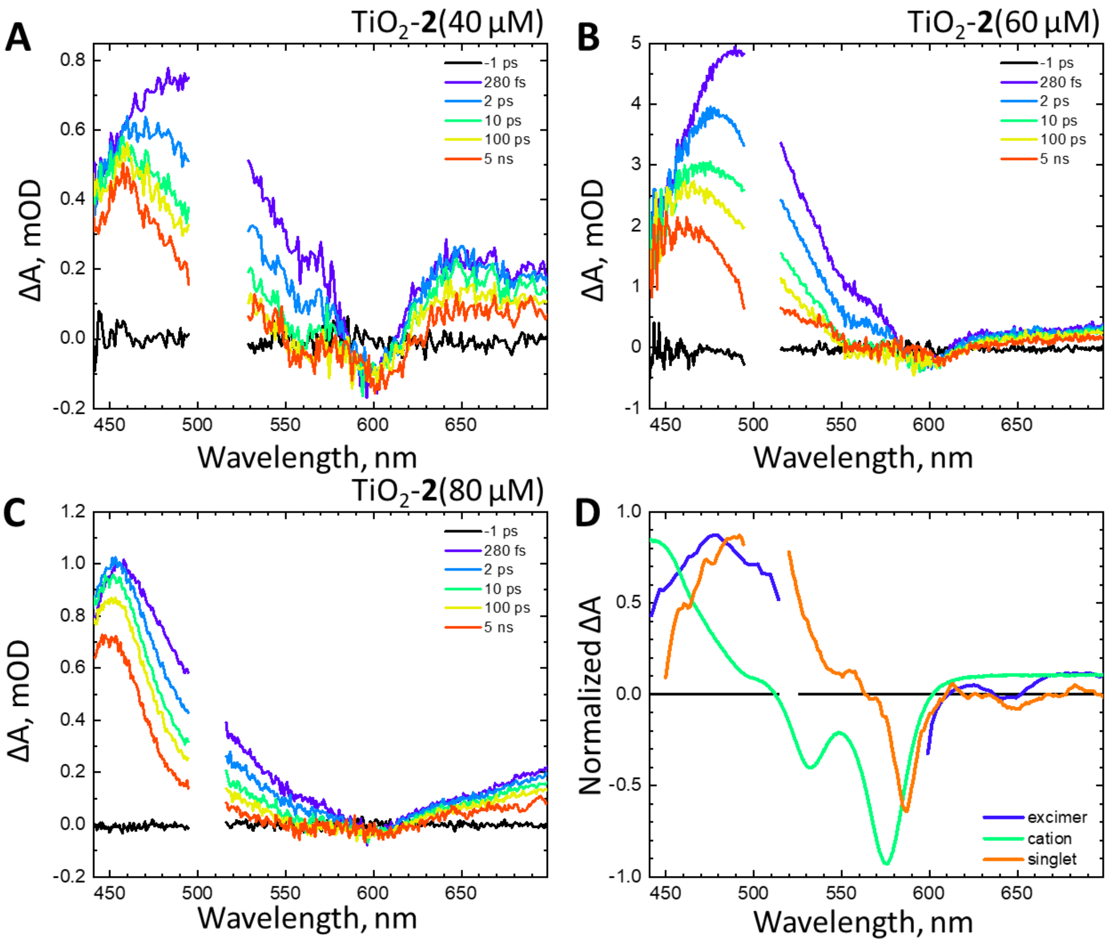

Figure S4. Transient absorption spectra of **A.** TiO_2-**2**(40 μM), B. TiO_2-**2**(60 μM), **C.** TiO_2-**2**(80 μM) after 515 nm (30 nJ/pulse) excitation. Pump-probe delays are shown in the legend. Note that the 60 μM has a slightly higher surface coverage than the 80 μM case, as shown in **Fig. 2B**. **D.** Comparison of the delta absorption signals of the singlet (orange) (determined via low concentration THF solution TA studies), cation (green) (determined via spectroelectrochemistry), and excimer (purple) (determined via high concentration THF solution TA studies).

Global Fit Results

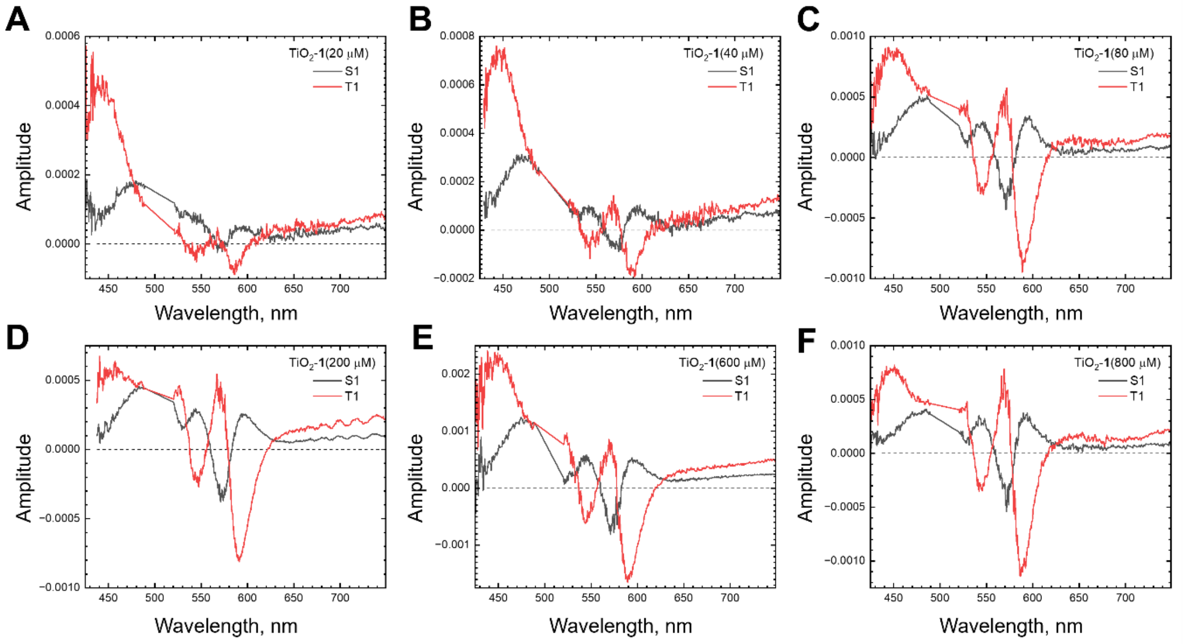

Figure S5. DAS for a 3-component parallel decay model for TiO_2-**1** with S_1 and T_1 representing the initial and final decaying states, respectively. A minor additional pathway was assumed to be related to cation formation. Loading solution concentrations are **A**. 20 µM, **B**. 40 µM, **C**. 80 µM, **D**. 200 µM, **E**. 600 µM, **F**. 800 µM. A (S1) and B(T1) were extracted, with the peak amplitudes determined at 460 nm for S1 and 565 nm for T1 for determine the amplitude ratios in Figure S6.

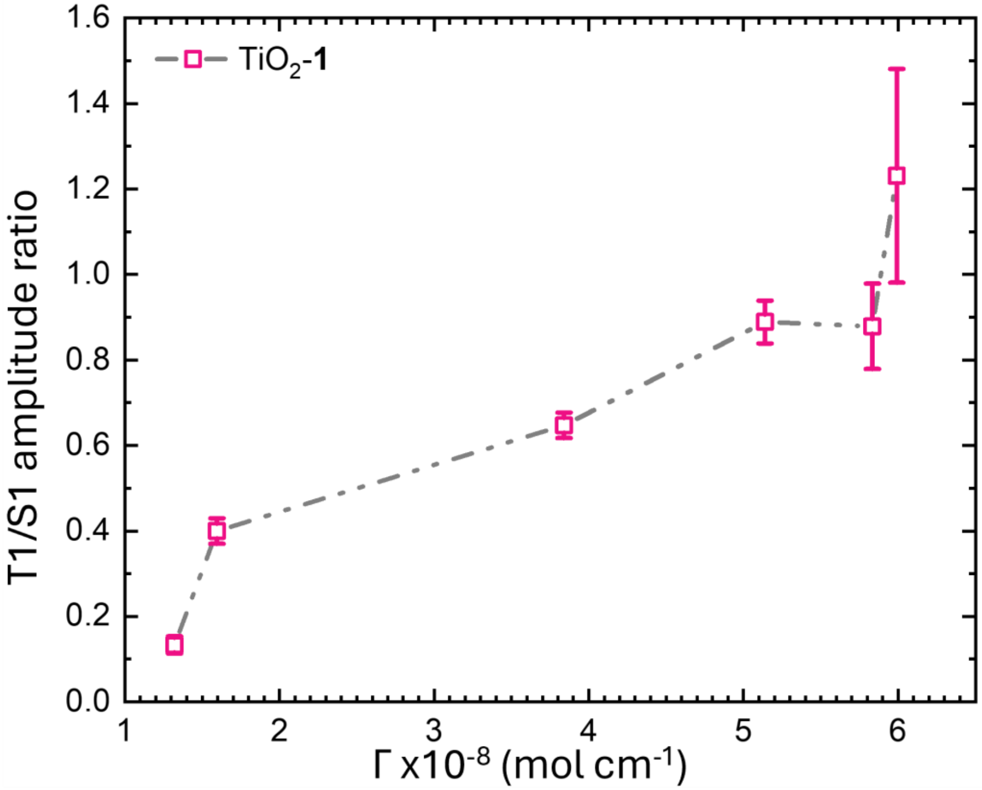

Figure S6. Relative yield of triplets based on global fit amplitudes of S1 and T1 in **Figure S5** plotted vs surface coverage. This method produces similar results to **Figure 3** in the main text showing a steady increase in triplet excited states with an increase in surface coverage.

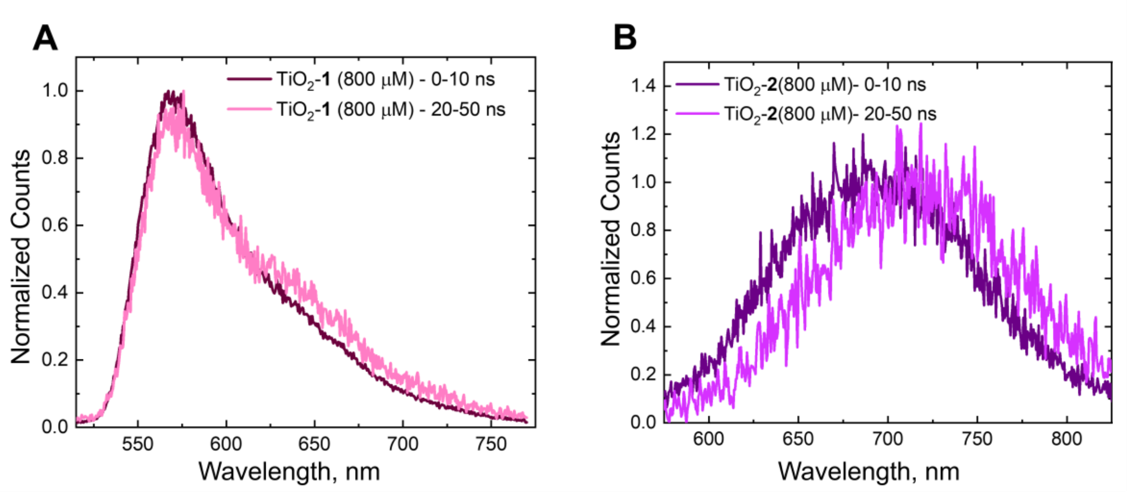

Figure S7. Time resolved photoluminescence data of **A**. TiO_2-**1** (800 µM) and **B**. TiO_2-**2** (800 µM) photoexcited at 525 nm in a 100 ns window at early (integrated for the first 10 ns, dark) and late (integrated over 20-50 ns, light).

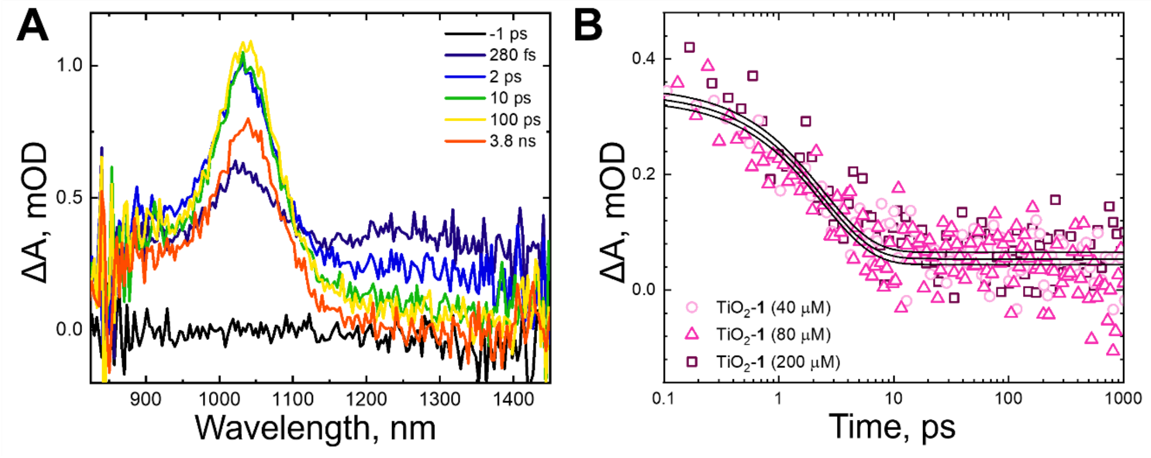

Figure S8. A. Transient absorption spectra of TiO_2-**1**(40 µM) after 515 nm (30 nJ/pulse) excitation. Pump-probe delays are shown in the legend. **B.** Normalized transient absorption kinetics at probe wavelengths of 1300 nm corresponding to the singlet excited state for TiO_2-**1**(40 µM) (light pink circles), TiO_2-**1**(200 µM) (dark pink squares). Fits shown as black lines and described in the text.

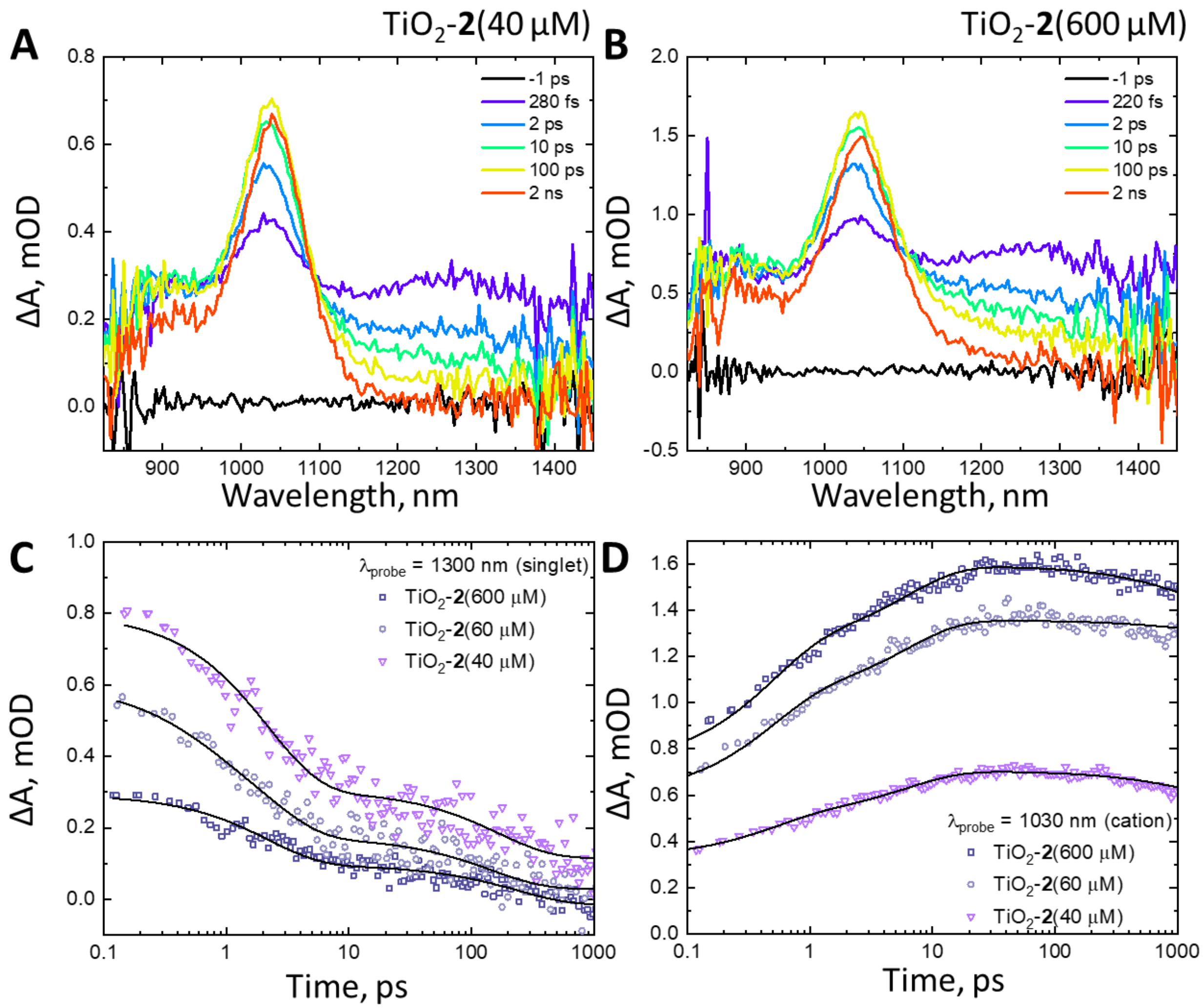

Figure S9. Transient absorption spectra of **A.** TiO_2-**2**(40 μM), **B**. TiO_2-**2**(600 μM) after 515 nm (30 nJ/pulse) excitation. Pump-probe delays are shown in the legend. Transient absorption kinetics at probe wavelengths of **C**. 1300 nm corresponding to the singlet excited state and **D**. 1030 nm corresponding to the cation for TiO_2-**2**(40 μM) (light purple triangles), TiO_2-**2**(60 μM) (purple circles), and TiO_2-**2**(600 μM) (dark purple squares). Fits shown as black lines and described in the text.

Table S1. Summary of time constants corresponding to the cation growth at λ_{probe} = 1030 nm for TiO_2-**1** and TiO_2-**2**. All measured surface coverages were fit to shared time constants.

	TiO_2-**1**<br>λ_{probe} = 1030 nm	TiO_2-**2**<br>λ_{probe} = 1030 nm
**A_1**	0.85	0.65
**τ_1, ps**	0.67 (±0.09)	0.98 (±0.1)
**A_2**	0.15	0.35
**τ_2, ps**	10.9 (±1.7)	7.8 (±0.7)

Table S2. Summary of time constants corresponding to the singlet excited state at λ_{probe} = 1300 nm for all measured surface loadings for TiO_2-**1** and TiO_2-**2**. All measured surface coverages were fit to shared time constants.

	TiO_2-**1**<br>λ_{probe} = 1300 nm	TiO_2-**2**<br>λ_{probe} = 1300 nm
**A_1**	0.6	0.7
**τ_1, ps**	0.84 (±0.32)	2.0 (± 0.12)
**A_2**	0.4	0.3
**τ_2, ps**	6.1 (±1.6)	163 (±18)

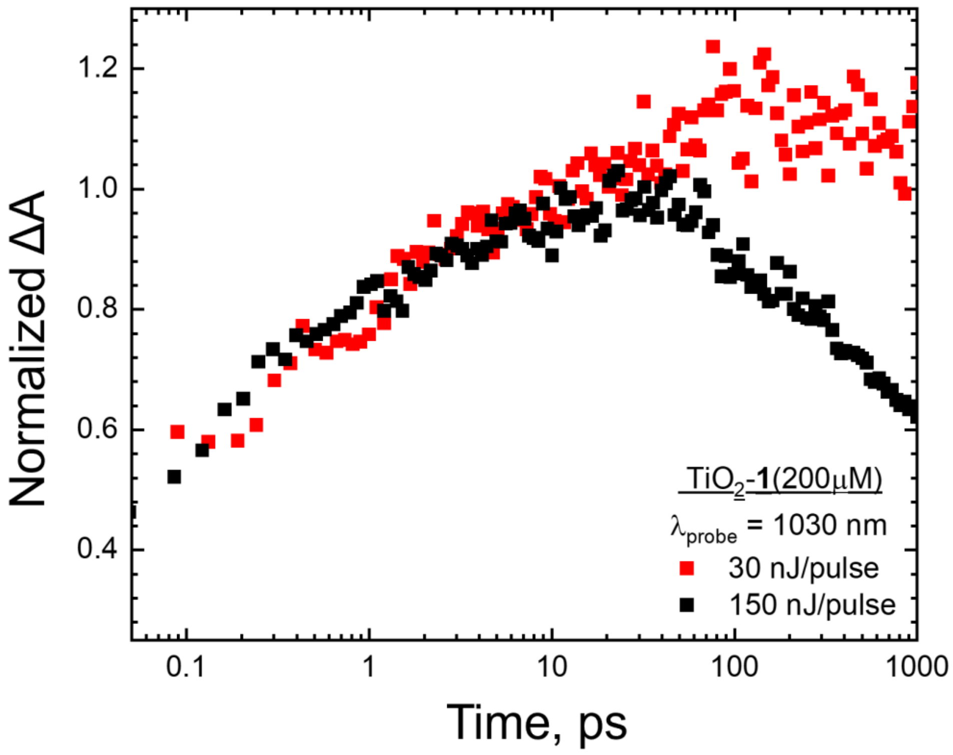

Figure S10. Normalized transient absorption kinetics of TiO_2-**1** (200 μM) at 1030 nm, where the cation signal appears, at two different excitation powers: 30 nJ(red) and 150 nJ (black).

Binding Simulations

A grid of 100x100 sites emulating a TiO_2 surface was constructed in MATLAB, and sites were randomly populated for different loadings from 1-90%.[1] The DBSCAN cluster analysis was performed on the result, yielding number of clusters of up to 8 (representing a full set of nearest neighbors in the unit cell). The occurrences as a function of loading were extracted and are shown in Figure S11.

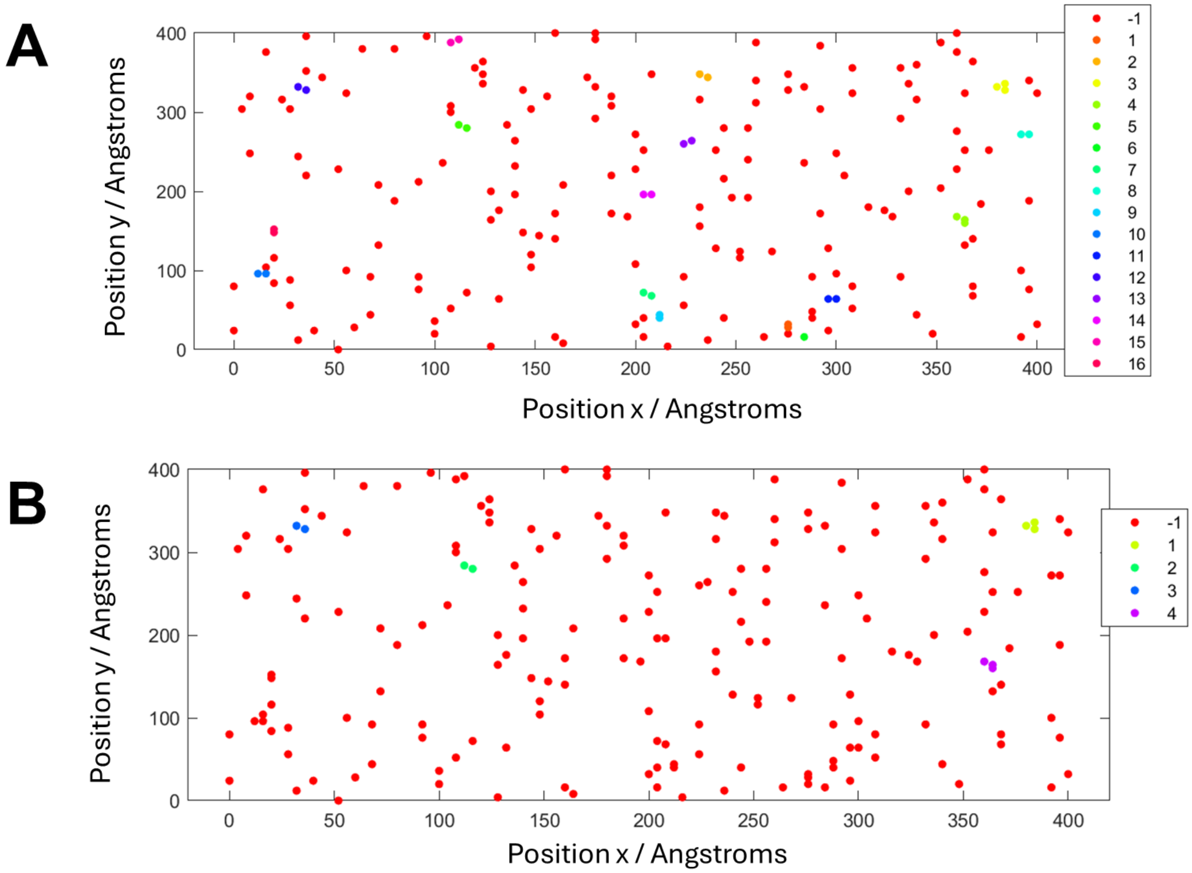

Figure S11. Simulation results for 2% dye occupation of a roughly 400x400 Angstrom lattice of TiO_2 sites (10000 sites). The occupation is generated by choosing random x and y positions within the lattice, with no cooperativity. Colors indicate clusters of (a) two and (b) three dyes identified through the DBSCAN algorithm in MATLAB.

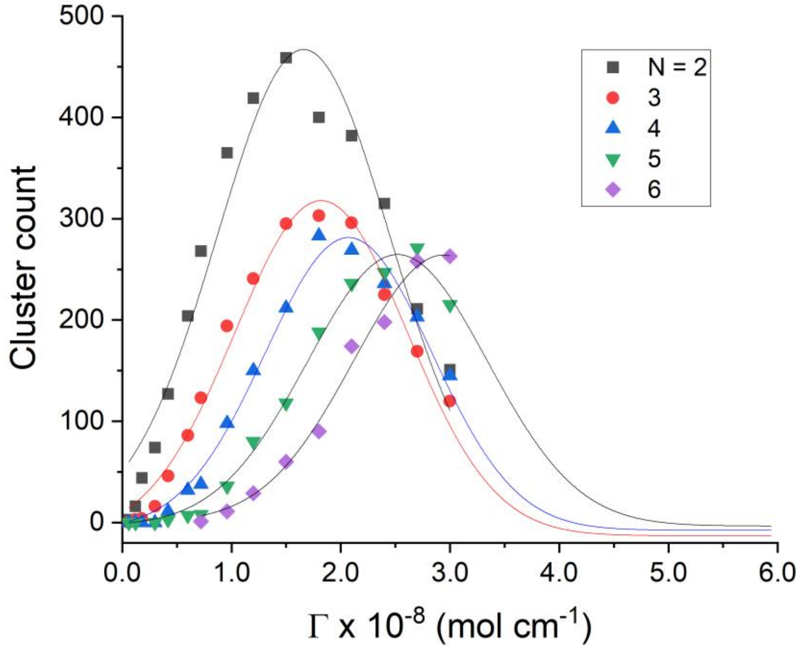

Figure S12. Occurrences for dimer (N=2) and larger clusters from DBSCAN analysis of a grid of 10000 sites with increasing loading. X-axis has been translated to surface coverage to match Figure 3 of the main text.
